# Increasing number of cases and outbreaks caused by *Candida auris* in the EU/EEA, 2020 to 2021

**DOI:** 10.2807/1560-7917.ES.2022.27.46.2200846

**Published:** 2022-11-17

**Authors:** Anke Kohlenberg, Dominique L Monnet, Diamantis Plachouras, Candida auris survey collaborative group, The Candida auris survey collaborative group includes the following national experts, Birgit Willinger, Katrien Lagrou, Ivva Philipova, Ana Budimir, Linos Hadjihannas, Markella Marcou, Lucie Bareková, Jan Kubele, Maiken Cavling Arendrup, Liidia Dotsenko, Laura Lindholm, Outi Lyytikäinen, Marie Desnos-Ollivier, Françoise Dromer, Jane Hecht, Oliver Kurzai, Lida Politi, Georgia Vrioni, Ágnes Hajdu, Katalin Kiss, Ólafur Guðlaugsson, Susanna Frost, Michela Sabbatucci, Ieva Voita, Esther Walser-Domjan, Rolanda Valintėlienė, Alexandre Mzabi, Monique Perrin, Rodianne Abela, Daan W Notermans, Paul E Verweij, Oliver Kacelnik, Miriam Sare, Katarzyna Dzierżanowska-Fangrat, Ana Lebre, José Artur Paiva, Gabriel Adrian Popescu, Roxana Serban, Slavka Litvová, Mária Štefkovičová, Mojca Serdt, Rok Tomazin, Ana Alastruey-Izquierdo, Erja Chryssanthou

**Affiliations:** 1 European Centre for Disease Prevention and Control (ECDC), Stockholm, Sweden; 2 The members of the *Candida auris* survey collaborative group are listed under Collaborators and at the end of the article

**Keywords:** Europe, healthcare-associated infections, fungal infections, multidrug-resistance, outbreak, surveillance, Candida

## Abstract

The number of cases of *Candida auris* infection or carriage and of countries reporting cases and outbreaks increased in the European Union and European Economic Area during 2020 and 2021. Eight countries reported 335 such cases in 2020 and 13 countries 655 cases in 2021. Five countries experienced outbreaks while one country reported regional endemicity. These findings highlight the need for adequate laboratory capacity and surveillance for early detection of *C. auris* and rapid implementation of control measures.

The European Centre for Disease Prevention and Control (ECDC) conducted two surveys collecting information on the epidemiological situation, laboratory capacity and preparedness for *Candida auris* in the European Union and European Economic Area (EU/EEA) for the periods 2013 to 2017 and January 2018 to May 2019 [1,2], but this information was not updated after the start of the COVID-19 pandemic. Attention to *C. auris* was raised again after a large outbreak affecting healthcare facilities in two regions in Italy [3], resulting in the initiation of a third *C. auris* survey in April 2022 to update the information on the epidemiological situation and control efforts for *C. auris* in the EU/EEA.

## Survey on the epidemiological situation, laboratory capacity and preparedness for *Candida auris*

The national focal points for healthcare-associated infections and their alternates were invited to complete the third *C. auris* survey on 4 April 2022. This survey included 14 questions on the aggregated number of cases of *C. auris* infection or carriage (in the following called *C. auris* cases) and outbreaks reported per year in the period from June 2019 to December 2021 (with the option to also add retrospectively identified cases for the period from January 2013 to May 2019), on the national capacity for laboratory identification and on preparedness for *C. auris*. The questions were the same as in the previous two surveys but included an additional question on the epidemiological stage (described below).

## Reported cases

**Figure 1:**
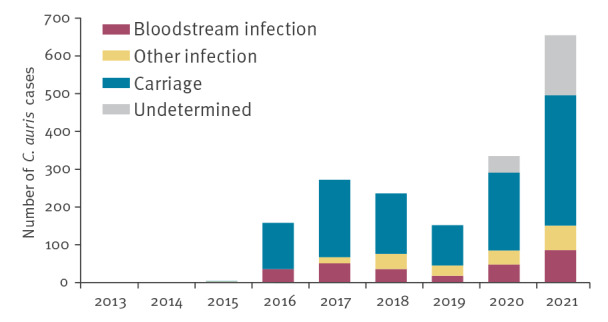
Reported cases of *Candida auris* infection or carriage, EU/EEA, 2013–2021 (n = 1,812)^a^

EEA: European Economic Area; EU: European Union.

^a^ Data reported by the United Kingdom until 2019 were excluded to ensure comparability over time by including the same set of countries. For this reason, the absolute case numbers differ from the number of cases reported for 2013–2019 in previous reports [1,2].

Replies to the survey were received from all 30 invited EU/EEA countries. Combining data from the three surveys, 1,812 *C. auris* cases were reported by 15 EU/EEA countries from 2013 to 2021. Case numbers by country and year are shown in Table 1. The number of reported cases nearly doubled between 2020 (335 cases reported by eight countries) and 2021 (655 cases reported by 13 countries) and were considerably higher than in previous years (Table 1, Figure 1). For most cases, carriage was reported (n = 1,146; 63.2%), while a bloodstream or another type of infection was reported for 277 (15.3%) and 186 (10.3%) cases, respectively. For the remaining 203 (11.2%) cases, no information on infection or carriage was available. Eleven EU/EEA countries had not detected any *C. auris* cases until 2021 and in four countries, information on *C. auris* cases was not available at national level (Table 1). In addition to the increase in the number of cases overall, the number of countries reporting *C. auris* cases increased, with a maximum of 13 countries reporting cases in 2021 (Table 1). Information on *C. auris* cases was collected for the period 2013 to 2021 in a standardised format. However, cases reported outside this period are mentioned in the footnotes to Table 1 if we believed that they represented a relevant change such as a new country affected or an earlier date of detection than previously known. 

**Table 1:** Reported cases of *Candida auris* infection or carriage, EU/EEA, 2013–2021 (n = 1,812)

Country	2013	2014	2015	2016	2017	2018	2019	2020	2021	2013–2021
Austria	0	0	0	0	0	1	0	2	1	4
Belgium	0	0	0	1	0	0	3	0	1	5
Bulgaria	0	0	0	0	0	0	0	0	0	0
Croatia	0	0	0	0	0	0	0	0	0	0
Cyprus	0	0	0	0	0	0	NA	NA	NA	NA
Czechia	0	0	0	0	0	0	1	0	0	1
Denmark	0	0	0	0	0	0	0	0	2	2
Estonia	0	0	0	0	0	0	0	0	0	0
Finland	0	0	0	0	0	0	0	0	1	1
France^a^	0	0	2	1	1	0	3	4	4	15
Germany	0	0	2	0	5	2	3	5	10	27
Greece	0	0	0	0	0	0	3	13	58	74
Hungary	0	0	0	0	0	0	0	0	0	0
Iceland	0	0	0	0	0	0	0	0	0	0
Ireland	0	0	0	0	0	0	0	0	1	1
Italy	NA	NA	NA	NA	NA	NA	1	49	242	292
Latvia	NA	NA	NA	NA	NA	NA	NA	NA	NA	NA
Liechtenstein	NA	NA	NA	NA	NA	NA	NA	NA	NA	NA
Lithuania	NA	NA	NA	NA	NA	NA	NA	0	0	0
Luxembourg	0	0	0	0	0	0	0	0	0	0
Malta	0	0	0	0	0	0	0	0	0	0
The Netherlands	0	0	0	0	0	2	1	1	1	5
Norway	0	0	0	1	0	1	0	0	2	4
Poland	NA	NA	NA	NA	NA	NA	2	0	0	2
Portugal^b^	NA	NA	NA	NA	NA	NA	NA	0	0	0
Romania	NA	NA	NA	NA	NA	NA	NA	NA	NA	NA
Slovakia	0	0	0	0	0	0	0	0	0	0
Slovenia	0	0	0	0	0	0	0	0	0	0
Spain	0	0	0	155	266	230	135	260	331	1,377
Sweden	0	0	0	0	0	0	0	1	1	2
**EU/EEA**	**0**	**0**	**4**	**158**	**272**	**236**	**152**	**335**	**655**	**1,812**

EEA: European Economic Area; EU: European Union; NA: information not available at national level.

^a^ France reported one case retrospectively identified in 2007 which is not included in this table [18].

^b^ Portugal reported one *C. auris* case for 2022 which is not included in this table.

Cells including one or more cases are coloured in grey for better visibility.

Information on the classification of cases of *C. auris* infection or carriage as imported or locally acquired was not available for 1,758 (97.0%) cases. Forty-four (2.4%) cases were reported as imported and 10 (0.6%) as locally acquired. A systematic analysis of the origin of imported cases was not possible due to scarce information. For the few cases with available information, countries in Africa (Egypt, Ethiopia, Kenya, South Africa), the Middle East (Iraq, Kuwait, United Arab Emirates) and Asia (India, Pakistan) were mentioned. Of note, there was also one cross-border transfer within the EU/EEA of a patient with *C. auris* originating from Spain.

## Reported outbreaks and epidemiological stage of dissemination

**Figure 2:**
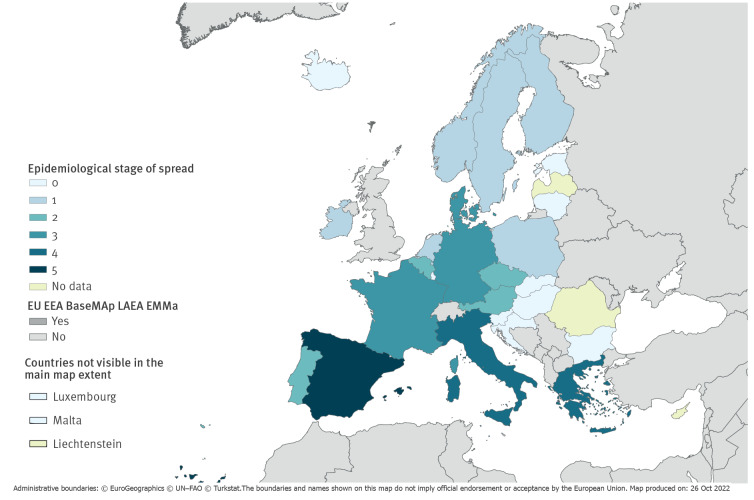
Epidemiological stage of *Candida auris* spread^a^, assessment by survey respondents in EU/EEA countries, 2022 (n = 30 countries)

EEA: European Economic Area; EMMa: European Centre for Disease Prevention and Control Map Maker tool; EU: European Union; LAEA: Lambert azimuthal equal-area projection.

^a^ Epidemiological stages of *C. auris* spread are defined as: Stage 0: No cases of *C. auris* infection or colonisation have been detected. Stage 1: Only imported cases of *C. auris* have been detected. Stage 2: Only sporadic cases of *C. auris* that were locally acquired or of unknown origin have been detected. Stage 3: Sporadic outbreaks of *C. auris* have occurred without or with only limited inter-facility spread. Stage 4: Multiple outbreaks of *C. auris* with verified or plausible inter-facility spread have occurred. Stage 5: *C. auris* is endemic in parts of the country (regional spread).

In the period 2019 to 2021, five countries (Denmark, France, Germany, Greece and Italy) reported 14 *C. auris* outbreaks defined as two or more cases with an epidemiological link, with 327 affected patients in total. The number of patients affected per outbreak ranged from two to 214 (Table 2). Inter-facility transmission occurred in eight outbreaks, and three outbreaks were reported as ongoing at the time of the survey (Table 2). The epidemiological stage of dissemination of *C. auris* was determined based on the respondents’ assessment in analogy to an epidemiological staging methodology that was previously developed and used for multidrug-resistant bacteria such as carbapenemase-producing Enterobacterales and carbapenem-resistant *Acinetobacter baumannii* [4,5]. Six countries reported that only imported *C. auris* cases had been detected (stage 1), four countries reported sporadic cases that were locally acquired or of unknown origin (stage 2), three countries reported sporadic outbreaks without or with only limited inter-facility spread (stage 3), two countries reported outbreaks with verified or plausible inter-facility spread (stage 4), and one country reported regional endemicity (stage 5) (Figure 2). This staging provides a snapshot of the epidemiological situation at the time of the survey and may not be indicative of the extent of future dissemination of *C. auris* within countries, especially within countries in stages 1–3 with currently few cases. 

**Table 2:** *Candida auris* outbreaks in EU/EEA countries detected in 2019–2021 (n = 14 outbreaks)

Outbreak	Year the outbreak was detected^a^	Number of cases	Duration (weeks)	Inter-facility transmission	Ongoing at time of survey
1	2019	214	149	Yes	Yes
2	2020	50	80	Yes	Yes
3	2020	15	44.5	Yes	No
4	2020	11	49	No	No
5	2021	10	5	Yes	No
6	2021	5	28	Yes	Yes
7	2021	4	3.5	Yes	No
8	2021	4	4	No	No
9	2021	3	4	Yes	No
10	2021	3	11	No	No
11	2021	2	8	No	No
12	2021	2	8	No	No
13	2021	2	39	Yes	No
14	2021	2	9	No	No
**Total**		**327**	**NA**	**8 yes**	**3 yes**

EEA: European Economic Area; EU: European Union; NA: not applicable.

^a^ The data for year signify only the year when the outbreak was detected, while the number of cases is reported for the whole duration of the outbreak (see column ‘Duration’).

## National surveillance, laboratory capacity and guidance

At the time of the survey, *C. auris* infection or carriage was notifiable in six of the 30 countries, prospective or retrospective surveillance was established in 12 countries, and 23 countries had a laboratory with reference capacity for identification and testing of *C. auris.* Twelve reference laboratories reported using matrix-assisted laser desorption/ionization time-of-flight mass spectrometry (MALDI-TOF MS) for identification of *C. auris*, 10 used MALDI-TOF MS in combination with other methods such as D1/D2 or Internal transcribed spacer (ITS) sequencing, and one reference laboratory reported using ITS sequencing only. Guidance for laboratory testing and for infection prevention and control was reported as available in 17 and 15 countries, respectively. These numbers represent a small improvement in preparedness and response compared with 2019 [2].

## Discussion

*Candida auris* is an emerging fungal pathogen that has caused outbreaks of invasive healthcare-associated infections worldwide [6]. *Candida auris* is frequently resistant to fluconazole, and multidrug-resistant and even pandrug-resistant *C. auris* isolates have also been described, thus leaving very few treatment options [6-8]. In Europe, the UK and Spain were the first countries to report outbreaks [9]. This survey showed that the number of *C. auris* cases increased in the EU/EEA as did the number of countries reporting cases and outbreaks for 2020 and 2021. Before these years, the number of cases with *C. auris* was mainly driven by a large outbreak in one country and had decreased in 2018 and 2019 after a peak in 2017. This situation changed in 2020 and 2021 when additional countries started to experience outbreaks. The role played by the coronavirus disease (COVID-19) pandemic in this increase is difficult to ascertain. Restricted travel may have decreased the risk of importation of *C. auris*. However, difficult-to-control outbreaks of *C. auris* have been reported in units caring for COVID-19 patients worldwide [10-13]. At least two of the *C. auris* outbreaks described in this report involved COVID-19 patients or units dedicated to the care of COVID-19 patients: the outbreak in Germany involving two cases occurred in a COVID-19 intensive care unit (ICU) [14], and an outbreak in Italy was amplified after introduction of *C. auris* into a COVID-19 ICU [3,15].

The results of this survey show that cases and outbreaks with *C. auris* occurred in several EU/EEA countries within only few years after the first cases had been reported in the EU/EEA. There is now evidence of inter-facility spread of *C. auris* in two EU/EEA countries, and *C. auris* was assessed as endemic in at least one region in one country, with cases no longer occurring as part of circumscribed outbreaks. Equally worrisome is the fact that for four countries, information at national level on whether *C. auris* cases occurred within the country was not available, raising the possibility of undetected transmission and outbreaks in the EU/EEA. The high proportion of cases without information on importation or local acquisition even in countries with available information highlights the need to improve follow-up and surveillance. Cases without a clear link to hospitalisation abroad are an indication of local acquisition and may represent the tip of the iceberg of undetected transmission. The reported interregional spread as well as regional endemicity in one country show that *C. auris* is in the process of establishing itself as a healthcare-associated pathogen in the EU/EEA, similar to other countries such as the United States [16]. European-level surveillance therefore needs to improve with case definitions and standardised and regular case-based reporting.

Despite the increase in the number of cases and difficult-to-control outbreaks, there are also examples from EU/EEA countries where transmission of *C. auris* was contained with control measures after the occurrence of only few cases, for example in Denmark and Germany [14,17]. National surveillance, a mycology reference laboratory that provides reference testing to hospital laboratories as well as national guidance for laboratory testing and infection control are basic elements required for the control of *C. auris*. More detailed options for response are described in the latest ECDC rapid risk assessment published in February 2022 [3].

## Conclusion

Local control of *C. auris* as soon as possible after introduction of a case to delay the establishment of *C. auris* in healthcare facilities will have a nationwide benefit for patients by reducing future healthcare-associated infections with *C. auris*. Control is more difficult to achieve once *C. auris* has spread within and between facilities or regions. It therefore continues to be of high importance that EU/EEA countries have adequate laboratory capacity and national surveillance for early detection of *C. auris* cases, and that measures to control and mitigate the consequences of its dissemination are rapidly implemented.

## The *Candida auris* survey collaborative group includes the following national experts

Birgit Willinger (Austria), Katrien Lagrou (Belgium), Ivva Philipova (Bulgaria), Ana Budimir (Croatia), Linos Hadjihannas, Markella Marcou (Cyprus), Lucie Bareková, Jan Kubele (Czech Republic), Maiken Cavling Arendrup (Denmark), Liidia Dotsenko (Estonia), Laura Lindholm, Outi Lyytikäinen (Finland), Marie Desnos-Ollivier, Françoise Dromer (France), Jane Hecht, Oliver Kurzai (Germany), Lida Politi, Georgia Vrioni (Greece), Ágnes Hajdu, Katalin Kiss (Hungary), Ólafur Guðlaugsson (Iceland), Susanna Frost (Ireland), Michela Sabbatucci (Italy), Ieva Voita (Latvia), Esther Walser-Domjan (Liechtenstein), Rolanda Valintėlienė (Lithuania), Alexandre Mzabi, Monique Perrin (Luxembourg), Rodianne Abela (Malta), Daan W. Notermans, Paul E. Verweij (Netherlands), Oliver Kacelnik, Miriam Sare (Norway), Katarzyna Dzierżanowska-Fangrat (Poland), Ana Lebre, José Artur Paiva (Portugal), Gabriel Adrian Popescu, Roxana Serban (Romania), Slavka Litvová, Mária Štefkovičová (Slovakia), Mojca Serdt, Rok Tomazin (Slovenia), Ana Alastruey-Izquierdo (Spain) and Erja Chryssanthou (Sweden).
